# Functional SNPs in *HSPA1A* Gene Predict Risk of Coronary Heart Disease

**DOI:** 10.1371/journal.pone.0004851

**Published:** 2009-03-31

**Authors:** Meian He, Huan Guo, Xiaobo Yang, Xiaomin Zhang, Li Zhou, Longxian Cheng, Hesong Zeng, Frank B. Hu, Robert M. Tanguay, Tangchun Wu

**Affiliations:** 1 Institute of Occupational Medicine and the Ministry of Education Key Lab of Environment and Health, School of Public Health, Tongji Medical College, Huazhong University of Science and Technology, Wuhan, Hubei, China; 2 Department of Cardiology, Union Hospital, Tongji Medical College, Huazhong University of Science and Technology, Wuhan, Hubei, China; 3 Department of Cardiology, Tongji Hospital, Tongji Medical College, Huazhong University of Science and Technology, Wuhan, Hubei, China; 4 Departments of Nutrition and Epidemiology, Harvard School of Public Health, Boston, Massachusetts, United States of America; 5 Laboratory of Cellular and Developmental Genetics, Department of Medicine, Faculty of Medicine, and CREFSIP, Pavilion C.E. Marchand, Université Laval, Québec, Canada; Karolinska Institutet, Sweden

## Abstract

**Background:**

HSP70 plays crucial roles in endothelial cell apoptosis, which is involved in the early phase and progress of coronary heart disease (CHD). However, the association between polymorphisms of *HSP70* genes and the risk of CHD still remains unclear. Our aim was to determine whether genetic variants in the *HSPA1A* gene are associated with the risk of CHD.

**Methodology/Principal Findings:**

By resequencing and genotyping, the associations of 2 single nucleotide polymorphisms (SNPs) +190G/C (rs1043618) and −110A/C (rs1008438) in the *HSPA1A* gene with risk of CHD were determined in a 1,003 pairs case-control study. The SNP function was further analyzed using a luciferase reporter assay in two cell lines. The results indicated that +190CC genotype was associated with significantly higher risk of CHD when compared with +190GG genotype (OR = 1.56, 95% CI: 1.10–2.20, *P* = 0.012), while association between −110A/C polymorphism and CHD was not statistically significant (*P*>0.05). However, the −110C/+190C haplotype had a significantly higher risk of CHD when compared with the −110A/+190G haplotype (OR = 1.17, 95% CI: 1.01–1.34, *P* = 0.031). Luciferase reporter assays showed that the +190C allele resulted in 14%∼45% reduction in luciferase expression in endothelial and non-endothelial cells when compared with the +190G allele.

**Conclusions/Significance:**

The identified genetic variants in the *HSPA1A* gene combinatorially contribute towards the susceptibility to CHD likely by affecting the level of synthesis of HSP70. This study may provide useful markers for identification of subjects at risk for CHD.

## Introduction

Coronary heart disease (CHD) is one of the leading causes of morbidity and mortality in China[1]. Each year there are about 2 million deaths from stroke- and CHD-related causes [2]. Gaining insights into the pathogenesis of CHD and identification of subjects at risk for CHD remains a tremendous challenge. It is well established that endothelial cells are the first protective barrier of vessel walls against various endogenous and exogenous stressors, and they also play crucial roles in controlling vascular tone, permeability, blood flow, coagulation, thrombolysis, inflammation and tissue repair[3]. Damage or apoptosis of endothelial cells is often considered as the initiating event of atherogenesis and also accelerates endothelialized plaque erosion and thrombosis[4], which will promote the development of acute cardiovascular incidents of CHD, such as unstable angina and myocardial infarction[5].

HSP70, as the main molecular chaperone, plays important roles in protecting against a wide variety of stress stimuli, including heavy metals, inflammation, and oxidative/ischemic injury[6]. Numerous evidences have corroborated the anti-oxidant and anti-apoptosis roles of HSP70[7]–[11]. HSP70 has also been found in the central portion of human atherosclerotic plaques[12]. Endothelial cells subjected to various stress conditions express increased amounts of HSP70 protein, which then functions as an antioxidant[8] and inhibits key processes of apoptosis pathways, to protect the integrity and functional activity of endothelial cells[13]. In addition, both *in vivo* and *in vitro* studies, indicated that the increased expression of HSP70 can protect the heart from stressful injury[14]–[17] and was associated with a reduction in myocardial apoptosis in ischemia-reperfusion injury[18]. It has been reported that the SNPs and haplotypes of *HSP70* may contribute to certain disease susceptibility and stress tolerance [19]–[25]. *HSPA1A* is the key component of the 12 members of the HSP70 family being expressed both under normal conditions and substantively stimulated after different stresses[26], [27]. Based on the important role of HSP70 in protecting the integrity and functional activity of endothelial cells and its anti-oxidant properties, we hypothesized that genetic variations in the *HSPA1A* gene might affect HSP70 protein expression, thus conferring one's predisposition to CHD.

To comprehensively evaluate the potential implications of 2 single nucleotide polymorphisms (SNPs) of *HSPA1A* gene (−110A/C and +190G/C polymorphisms) in the etiology of CHD, we conducted a large-scale case-control study of 1,003 CHD cases and 1,003 age- and sex- frequency matched controls in a Chinese population. Using an *in vivo* reporter assay in endothelial cells (HUVEC) and non-endothelial cells (Hela), we found that the SNP +190G/C (dbSNP accession number *rs1043618*), located in the 5′ untranslated region (UTR) of *HSPA1A* gene, may affect the synthesis level of Hsp70 protein through translation efficiency or post-transcriptional regulation.

## Results

### SNPs Identification in the 5′ Flanking Region of *HSPA1A* Gene in Han Chinese

Resequencing of the *HSPA1A* gene in 60 unrelated Han Chinese revealed two SNPs in 5′ flanking region of *HSPA1A* gene. Referring the gene transcription start site as +1, one SNP is −110A/C (dbSNP accession number *rs1008438*) in the core promoter region and the other is +190G/C in the 5′UTR region, with minor allele frequency of 0.355 and 0.240 respectively. There were significant differences in allele frequencies of −110A/C and +190G/C across different ethnic groups (*P*<0.05) (Table 1).

**Table 1:** Comparison of Allele Frequencies of Polymorphisms in *HSPA1A* genes among Different Populations [36].

SNPs	Population	Wild type	Mutation type	*P* Value
*HSPA1A* −110A/C		A	C	0.000
	Han Chinese	0.645	0.355	
	Sub-Saharan African	0.083	0.917	
	Hispanic	0.477	0.523	
	European	0.886	0.114	
*HSPA1A* +190G/C		G	C	0.001
	Han Chinese	0.760	0.240	
	Japanese	0.802	0.198	
	CEU*	0.658	0.342	
	Yoruba	0.902	0.098	

* CEU: Utah residents with ancestry from northern and western Europe.

### General Characteristics of the Subjects

The clinical and demographic features of subjects have been described in our previous study[28]. In summary, CHD patients had a higher prevalence of conventional vascular risk factors, including smoking, non-drinking, history of hypertension and DM, family history of CHD and higher level of FBG, whereas TC level in patients were lower in cases than controls, probably due to cholesterol-lowering treatment in the cases.

### *HSPA1A* Genotypes and CHD Risk

These two bi-allelic SNPs were investigated in 1,003 CHD patients and in controls. The distributions of SNPs +190G/C and −110A/C did not depart from the Hardy-Weinberg equilibrium in the control group (*P* = 0.18 and 0.32 respectively). Genotype frequencies of the two studied SNPs are summarized in Table 2. There was borderline significant difference in genotype distributions of SNP +190G/C between CHD cases and controls, but adjustment for the conventional risk factors such as age, sex, pack-year of smoking, drinking, activity, hypertension, DM, and family history of CHD yielded significant results (*P* = 0.012). Compared with GG genotype of +190G/C, subjects with CC genotype had a higher risk of CHD after adjusting for the conventional risk factors above (Crude OR = 1.33, 95% CI: 1.00–1.77, *P* = 0.052 and adjusted OR = 1.56, 95% CI: 1.10–2.20, *P* = 0.012 respectively). There was no significant difference between CHD cases and control group in −110A/C locus before or after adjusting for conventional risk factors (*P*>0.05). Haplotype analysis indicated that patients with −110C/+190C haplotype had a higher risk of CHD when compared with −110A/+190G haplotype (OR = 1.17, 95% CI: 1.01–1.34, *P* = 0.031) (Table 3).

**Table 2:** Analysis of Association between *HSPA1A* Polymorphisms and Risk of CHD in a Chinese population.

Genotype	Cases		Controls		Crude OR (95% CI)	Adjusted OR (95% CI)*
	n	%	n	%		
+190G/C						
GG	434	44.2	479	47.8	1.00	1.00
GC	416	42.4	415	41.4	1.11(0.92–1.34)	1.04(0.83–1.31)
CC	131	13.4	109	10.8	1.33(1.00–1.77)†	1.56(1.10–2.20)‡
GC+CC	547	55.8	524	52.2	1.15(0.97–1.38)	1.13(0.91–1.40)
−110A/C						
AA	317	32.3	350	34.9	1.00	1.00
AC	476	48.6	472	47.1	1.11(0.91–1.36)	1.15(0.90–1.46)
CC	187	19.1	181	18.0	1.14(0.88–1.47)	1.17(0.86–1.60)
AC+CC	663	67.7	653	65.1	1.12(0.93–1.35)	1.14(0.91–1.43)

* Adjusted for age, sex, pack-year of smoking, drinking, activity, hypertension, DM and family history of CHD. Compared with GG genotype.

† *P* = 0.052 and

‡ *P* = 0.012, respectively.

**Table 3:** Haplotype Distribution of *HSPA1A* in CHD and Control Group.

Haplotype (−110/+190)	CHD		Controls		OR (95% CI)
	n	%	n	%	
−110A/+190G	1092	54.44	1118	55.74	1.00
−110C/+190C	657	32.75	577	28.76	1.17 (1.01–1.34)*
−110C/+190G	208	10.37	256	12.76	0.83 (0.68–1.02)
−110A/+190C	49	2.44	55	2.74	0.91 (0.62–1.35)
Total	2006	100	2006	100	—

Polymorphic bases were in 5′ to 3′ order and from left to right the order is −110A/C and +190G/C.

* Compared with −110A/+190G haplotype, *P* = 0.031.

### *HSPA1A* 5′ Flanking Region Carrying the +190C Polymorphisms leads to Lower HSP70 Expression

**Figure 1:**
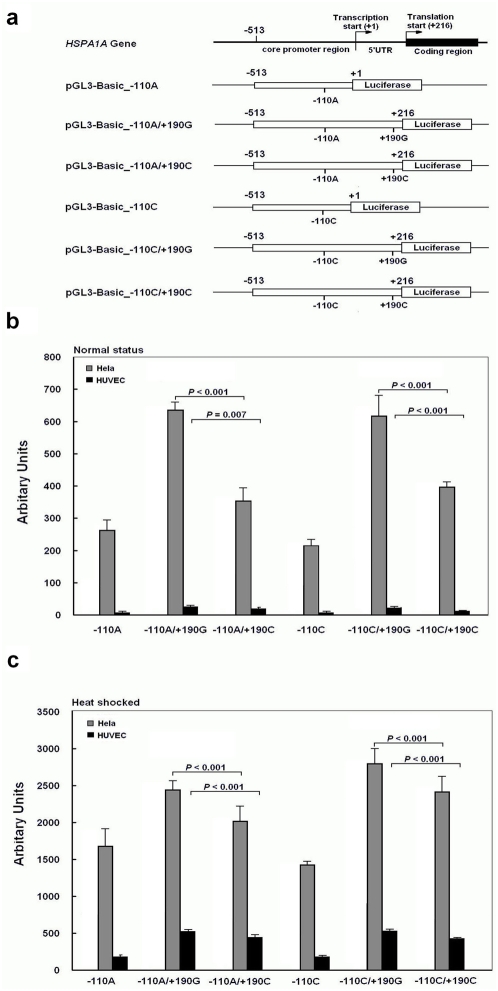
Effects of −110A/C and +190G/C polymorphisms on luciferase activity. (a), Schematic representation of *HSPA1A* gene 5′ flanking region and reporter constructs. (b) to (c), the six reporter constructs and empty pGL3-Basic was transfected into Hela and HUVEC cells under normal culture (b) or heat shocked conditions (c). All constructs were cotransfected with pRL-SV40 to standardize the transfection efficiency. Fold increase of luciferase activity was measured by defining the activity of the empty pGL3-Basic vector as 1. The RLA values are presented as means, and the T bars represent standard deviations (SD). Mean±SD from triplicates.

**Figure 2:**
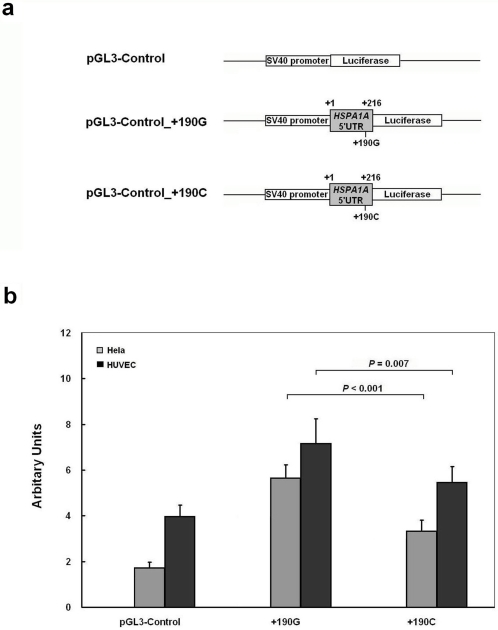
Effect of +190G/C polymorphism on the expression of luciferase. (a), Schematic representation of reporter constructs. (b), These constructs and empty pGL3-Control plasmid were transfected into Hela and HUVEC cells. All constructs were cotransfected with pRL-SV40 to standardize the transfection efficiency. RLA was determined 24 hours later in normal culture condition. The RLA values are presented as means, and the T bars represent standard deviations (SD). Mean±SD from triplicates.

To investigate the possible functional significance of the SNPs in the *HSPA1A* gene, plasmids were constructed with luciferase as reporter gene and transfected in cultured cells. Under normal culture status, the RLA of all constructs containing fragments that include both core promoter region and 5′UTR of *HSPA1A* gene (−513 to +216) were remarkably higher than those carrying only the core promoter region (−513 to −1) in Hela, and HUVEC cells (*P*<0.0001) (Figure 1b). As for the four distinct haplotype plasmids, the RLA of −110A/+190G and −110C/+190G haplotype were significantly higher than that of −110A/+190C and −110C/+190C respectively (*P*<0.001), but no differences were found between −110A/+190G and −110C/+190G haplotype nor between −110A/+190C and −110C/+190C haplotype, suggesting that the reduction of RLA was due to the presence of the +190C allele (Figure 1b). To further explore the regulatory properties of the SNPs, transfected cells were submitted to a heat shock. The RLA recorded, were increased for about 6∼7 times in Hela cells and about 20 times in HUVEC cells. In the heat shock groups, the results were similar to those under normal conditions in all cells (Figure 1c). The *HSPA1A* 5′-flanking region bearing the −110C/+190C haplotype exhibited about 30%∼50% lower RLA than the −110A /+190G haplotype in endothelial (HUVEC) and non-endothelial cells (Hela). We also inserted the full-length 5′UTR containing either +190G or +190C directly following the SV40 promoter and before the translation start codon of the firefly luciferase gene of pGL3-Control vector (Figure 2a). The full-length 5′UTR of *HSPA1A* gene increased luciferase activity of the pGL3-Control vector (*P*<0.001), with the +190C allele resulting in 14%∼45% reduction in luciferase expression compared to the +190G allele in Hela and HUVEC cell lines (*P*<0.001, and *P* = 0.007, respectively) (Figure 2b).

## Discussion

Our study is among the few that examined the association between SNPs of *HSPA1A* gene and risk of CHD. Resequencing of this gene in 60 unrelated Han Chinese revealed two SNPs in 5′ flanking region of *HSPA1A* gene; one is −110A/C in the core promoter and the other is +190G/C located in the 5′UTR region. The allele frequencies of these two SNPs in the Han Chinese population were significantly different from Sub-Saharan African, Hispanic and European races. Our large case-control analysis showed no strong association between −110A/C polymorphism and CHD, which was consistent with the result of a previous European study that indicated no association between this polymorphism and myocardial infarction[28]. However, we found that the +190C allele and the haplotypes containing +190C were significantly associated with CHD risk. Since the two SNPs both reside in the highly conserved regulatory region of the gene, we further explored their putative effects on *HSPA1A* promoter activity and found that the *HSPA1A* 5′UTR enhanced expression of a luciferase reporter driven either by the *HSPA1A* or SV40 promoter; the allele-specific data of reporter assays in both endothelial (HUVEC) and non-endothelial (Hela) cells yielded consistent results that the +190C allele in the 5′UTR of *HSPA1A* gene leads to a reduction in promoter activity and probably lower synthesis level of HSP70 protein than +190G allele.

The function of *HSPA1A* 5′UTR sequence of *HSPA1A* mRNA was comprehensively analyzed and reported to act as a general enhancer of mRNA translation[29]. Our findings are consistent with the above study and the effect of *HSPA1A* 5′UTR was independent of the promoter used. The *HSPA1A* 5′UTR sequence has an internal ribosome entry site (IRES) for translation initiation by a cap-dependent mechanism, which depends on its 3-dimensional secondary structures of full-length 5′UTR, especially the base-pairing status and nucleotide context of the 3′-terminal AUG-proximal region of *HSPA1A* 5′UTR [30]. Theoretical secondary structure folding analysis by Mfold program suggested that the *HSPA1A* 5′UTR sequence containing +190G or +190C allele had distinct thermodynamic parameters and different stem-loop structure in its 3′-terminal region[31]. Albeit untested experimentally, the modulation caused by the +190 G/C polymorphism, which is just 26 bp upstream the initial codon, may arise from altered stem-loop structure of the 5′UTR of *HSPA1A* mRNA, thus confer different efficiency of *HSPA1A* mRNA translation. Since heat shock treatment is the typical model up-regulating expression of HSP70, we can postulate that such difference in 5′UTR may also be applicable under other stresses, such as inflammation, ischemic and oxidative injury, all of which play critical roles in the pathogenesis of CHD.

Thus we showed that the −110C/+190C haplotype, which was significantly associated with CHD risk had a 30%∼50% lower RLA than −110A /+190G haplotype in both endothelial and non-endothelial cells. The RLA of *HSPA1A* promoter plasmid driven by −110A was higher than that of −110C in Hela cell lines, but didn't reach the significant level in HUVEC cells (data not shown). We would like to speculate that the −110A/C polymorphism is also a potential functional SNP, and that when combined with +190G/C polymorphism, may act as an additive effect on the synthesis of HSP70 protein.

The present study has several strengths. First, our population was highly homogenous with respect to ethnicity and geographic regions, which minimizes the possible biases related to population stratification. In addition, the large number of cases and controls provided sufficient power to detect moderate effects of the genotypes and haplotypes. Finally, the possible patho-physiological significance of the +190G/C polymorphism was further confirmed by detailed functional assays in both endothelial and non-endothelial cell lines with consistent results. This strengthened the association between the *HSPA1A* gene polymorphisms and CHD revealed by the present study. However two limitations should also be acknowledged. First, because this was a retrospective study, a possible selection bias (inclusion of patients surviving CHD) and/or systemic error may exist. However, there was no evidence that these SNPs influence survival. Meanwhile, we selected controls that had normal electrocardiograms and no history and signs of CHD, but we can not exclude the possibility that some of them were affected by silent myocardial infarction because we did not perform coronary angiography on those control subjects. However, the prevalence of CHD in China is still low[32], especially among those without positive ECG test or clinical symptoms. All our controls were required to have normal ECG and no clinical symptoms before enrollment, thus the false negative cases in the controls are likely to be rare.

In conclusion, our results of the large-scale case-control study and reporter assays support the hypothesis that reduced expression of HSP70 protein induced by the *HSPA1A* +190C allele may dampens its cytoprotection role on endothelial cells and thus contributes to one's predisposition to CHD. Our study provides the first evidence that genetic variants in *HSPA1A* gene may potentially contribute to the susceptibility to CHD. However, our findings need to be replicated in additional studies, especially in large prospective cohort studies.

## Materials and Methods

### Human Subjects and General Characteristics

The study design for this investigation has been described elsewhere[33]. Briefly, the study population was composed of 1,003 cases and 1,003 age- and sex- frequency matched controls. All enrolled subjects were unrelated ethnic Han Chinese. Case patients, who were enrolled from three hospitals (Tongji Hospital, Union Hospital, and Wugang Hospital) in Wuhan City, Hubei province, were diagnosed as having CHD according to WHO criteria or by coronary angiography (significant coronary artery stenoses ≥50% in at least one major coronary artery). A total of 1,078 patients diagnosed as having CHD were recruited; 1,003 of them (93.0%) consented to participate in the study and provided questionnaire information and blood samples. The control subjects, residing in the same communities as the cases, were judged to be free of CHD and peripheral atherosclerotic arterial disease by medical history, clinical examinations, and electrocardiography (ECG). The response rate for the controls was 92.4% (1,003 of 1,085). Subjects with severe liver and/or kidney disease were excluded. Subjects were classified as nonsmokers, former, or current smokers. Pack-years were calculated by multiplying the number of packs of cigarettes smoked per day by the number of years the person had smoked. BMI was calculated as weight in kilograms divided by the square of height in meters. Subjects were considered to be hypertensive if their systolic blood pressure was ≥140 mmHg and/or diastolic pressure ≥90 mmHg or they were already being treated with antihypertensive drugs. Medical history, socio-demographic information, family history of cardiovascular disease, medication use, home environment, and lifestyle factors were obtained through questionnaire interview. All subjects gave written consent after receiving a full explanation of the study. The Ethics Committee of Tongji Medical College approved the study.

### Screening for SNPs in 5′ Flanking Region of *HSPA1A* Gene in Chinese

DNA samples extracted from whole blood of 60 randomly selected healthy subjects were used to identify SNPs in the 5′ flanking region of *HSPA1A* gene (GenBank accession no. NM_005345) in the Han Chinese population. Sequencing reactions were carried out on an ABI 3100 genetic analyzer. All primers and reaction conditions are listed in Table S1.

### Genotyping of *HSPA1A* polymorphisms

Genotyping of *HSPA1A* was performed with 5′ nuclease TaqMan allelic discrimination assay on an ABI 7900HT real-time quantitative PCR system (Applied Biosystems), in 384-well format. For −110A/C polymorphism, the TaqMan primers were 5′- GCCTCTGATTGGTCCAAGGAA-3′ and 5′-GCTGCCAGGTCGGGAATAT-3′,while probes were FAM - AGGCGAAACCCCTGG-MGB for −110C and VIC- AGGCGAAAACCCTGG –MGB for −110A. The catalog number for +190G/C polymorphism was C-11917510-10. Finally, genotyping failed in 22 (2.20%) cases in +190G/C locus, 23 (2.30%) cases in −110A/C locus owing to DNA quantity or quality.

### Biological variables determination

Fasting blood glucose (FBG), total cholesterol (TC), and triglyceride (TG) were assayed using standard laboratory procedures at the Department of Clinical Laboratory at the Wuhan Union Hospital.

### Construction of Reporter Plasmids

We constructed six reporter plasmids based on pGL3-Basic vector (Promega, Madison, Wisconsin, USA), including four fragments compassing from −513 to +216 according to their haplotypes (−110A/+190G, −110A/+190C, −110C/+190G and −110C/+190C, respectively) and two core promoter regions from −513 to −1 containing either −110A or −110C allele. The position “+1” indicates the transcription start site (Figure 1a). They were all inserted into *Kpn* I/*Hind* III enzyme sites of pGL3-Basic. We also inserted the full-length 5′UTR containing +190G or +190C allele into the *Hind* III /*Nco* I enzyme sites of pGL3-Control vector. Primer pairs designed for plasmid constructs and site-specific mutagenesis are listed in Table S2. The direction and sequence authenticity of the above constructs were validated by restriction analysis and direct sequencing.

### Transient Transfection and Luciferase Reporter Assays

Hela cells were cultured with RPMI 1640 containing 10% fetal bovine serum and seeded into 96-well plates at a density of 3×10^4^ cells per well respectively, while 4×10^4^ human umbilical vein endothelial cells (HUVEC) were seeded into 48-well plates. Twenty-four hours later when cells had grown to about 70% confluence, each well was co-transfected with 100 ng pGL3-Basic plasmids or its constructs (defined as −110A, −110A/+190G, −110A/+190C, −110C, −110C/+190G, −110C/+190C) and 1 ng pRL-SV40 (Promega) using Lipofectamine 2000 (Invitrogen, Carlsbad, California, USA) according to the manufacturer's protocol. The Hela and HUVEC cells were harvested 24 hours after transfection, whereas in the heat-shocked group, cells were placed at 42°C for 1 hour in a water bath and then allowed to recover at 37°C for 4 hours before measurement. Then luciferase activity was measured using Dual-Luciferase Reporter Assay System (Promega) on a TD-20/20^n^ luminometer (Turner Design, Promega). Each construct was tested in six assays and the transfection experiments were performed three times independently. As for the pGL3-Control and its constructs, the same transfection procedures were done. The result was denoted as relative luciferase activity (RLA) since the luciferase activity was normalized by *Renilla* activity and the empty pGL3-Basic or pGL3-Control vector.

### Statistical Analysis

Normal distribution of data was checked by the Komogorov-Smirnov normality test. Data with a normal distribution were compared by Student's *t*-test, and those with unequal variance or without a normal distribution were analyzed by a nonparametric Mann-Whitney rank sum test. Different RLA of four haplotypes contained plasmids were determined by one-way ANOVA and Student-Newman-Keuls test. A Chi-square test was applied to compare categorical variables and the Hardy-Weinberg equilibrium of the polymorphisms. The linkage relationship between the two SNPs in *HSPA1A* genes was measured by linkage disequilibrium analyzer (LDA) program[34]. All genotype data for each sample were taken to infer the haplotypes by using the PHASE 2.0 program[35], a software for reconstruction of haplotypes from population genotype data by Bayesian statistical method. The associations between variants and CHD risk were estimated by odds ratios (ORs) and 95% confidence intervals (CIs) by using the unconditional logistic regression analyses with adjustment for multiple cardiovascular risk factors such as age, sex, pack-year of smoking, drinking activity, hypertension, diabetes mellitus (DM), and family history of CHD. A *P* value of less than 0.05 was considered significant. All data analyses were carried out with statistical analysis software package SPSS 12.0 (SPSS Inc., Chicago, Illinois, USA).

## Supporting Information

Table S1 (0.04 MB DOC)

Table S2 (0.03 MB DOC)
