# Supplementary material for: Functional SNPs in HSPA1A Gene Predict Risk of Coronary Heart Disease
Source: PLoS One. 2009 Mar 31;4(3):e4851. doi: 10.1371/journal.pone.0004851 (PMC2659421; doi:10.1371/journal.pone.0004851)
Supplement: Table S1 — (0.04 MB DOC) [file pone.0004851.s001.doc]

**Supplementary Table 1 Primer sequences used in resequencing of *HSPA1A* gene**

| **Primer name** | **Primers sequence ( 5’ to 3’ )** | **T_m_**  **(**°C**)** | **Annealing temperature (**°C**)** | **Amplification scope** | **Length of product**  **(bp)** |
| --- | --- | --- | --- | --- | --- |
| Primer1-foward | AGACATCAGCCTCCACACTTG | 60.0 | 58.0 | -1637 | 508 |
| Primer1-reverse | TCAGCCTTCCGAGTAGCAG | 59.7 |  | -1130 |  |
| Primer2-foward | CTGCTACTCGGAAGGCTGA | 59.7 | 58.5 | -1148 | 401 |
| Primer2-reverse | GAGGTATGTGGGCGTTGG | 59.6 |  | -748 |  |
| Primer3-foward | CCAAGTGCTCCTCCTACC | 59.6 | 57.5 | -792 | 460 |
| Primer3-reverse | ATTGTGGGGGCTTGCTGG | 59.7 |  | -333 |  |
| Primer4-foward | AGCCCCCTGCCCACAA | 59.2 | 58.5 | -376 | 472 |
| Primer4-reverse | CCGCACAGGTTCGCTCT | 59.4 |  | +95 |  |
| Primer5-foward | CCCTCCAGTGAATCCCAGAA | 59.9 | 60.5 | -214 | 491 |
| Primer5-reverse | CACCCCCACGCAGGAGTA | 61.9 |  | +276 |  |
