# Supplementary material for: Functional SNPs in HSPA1A Gene Predict Risk of Coronary Heart Disease
Source: PLoS One. 2009 Mar 31;4(3):e4851. doi: 10.1371/journal.pone.0004851 (PMC2659421; doi:10.1371/journal.pone.0004851)
Supplement: Table S2 — (0.03 MB DOC) [file pone.0004851.s002.doc]

**Supplementary table 2 Primer sequences used in reporter plasmids construction**

| **Purpose** |  | **Sequence ( 5’ to 3’ )** |
| --- | --- | --- |
| **pGL3-Basic based constructs**  **(5’-*Kpn* I, 3’-*Hind* III)** | -513 to +216 | TAGGGGTACCGACGGCTCCAACTCAGTAATC  ATGCAAGCTTGCCGGTTCCCTGCTCTCTGTCG |
|  | -513 to -1 | TAGGGGTACCGACGGCTCCAACTCAGTAATC  ATCCAAGCTTCCGGACCGCTTGCCCCTGGGCTT |
| **pGL3-Control based constructs**  **(5’- *Hind* III, 3’- *Nco* I)** | +1 to +216 | ATGCAAGCTTATAACGGCTAGCCTGAGGAGCTGC  ACTGCCATGGGCCGGTTCCCTGCTCTCTGTCG |
| **Site-specific mutagenesis** | -110A to C | GGACGGGAGGCGAAACCCCTGGAATATTCCC  GGGAATATTCCAGGGGTTTCGCCTCCCGTCC |
|  | +190G to C | CCCCAATCTCAGAGCCGAGCCGACAGAGAGC  GCTCTCTGTCGGCTCGGCTCTGAGATTGGGG |
